# An Oculist’s Test

**Published:** 1889-01

**Authors:** 


### AN OCULIST'S TEST.

In a large factory in which were employed several hundred persons, one of the workmen, in wielding his hammer, carelessly allowed it to slip from his hand. It flew half-way across the room and struck a fellow-workman in the left eye. The man averred that his eye was blinded by the blow, although a careful examination failed to reveal any injury, there being not a scratch visible. He brought a suit in the courts for compensation for the loss of half of his eyesight, and refused all offers of compromise. Under the law the owner of the factory was responsible for an injury resulting from an accident of this kind, and although he believed that the man was shamming, and that the whole case was an attempt at swindling, he had about made up his mind that he would be compelled to pay the claim. The day of the trial arrived, and in open court an eminent oculist retained by the defence, examined the alleged injured member, and gave as his opinion that it was as good as the right eye. Upon the plaintiff's loud protest of his inability to see with his left eye, the oculist proved him a perjurer, and satisfied the court and jury of the falsity of his claim. And how do you suppose he did it? Why, simply by knowing that the colors green and red combined make black. He prepared a black card on which a few words were written with green ink. Then the plaintiff was ordered to put on a pair of spectacles with two different glasses, the one for the right eye being red and the one for the left eye consisting of ordinary glass. Then the card was handed him and he was ordered to read the writing on it. This he did without hesitation, and the cheat was at once exposed. The sound right eye, fitted with the red glass, was unable to distinguish the green writing on the black surface of the card, while the left eye, which he pretended was sightless, was the one with which the reading had to be done.—*Pottery Gazette.*

